# Effects of temperature and photoperiod on daily activity rhythms of *Lutzomyia longipalpis* (Diptera: Psychodidae)

**DOI:** 10.1186/1756-3305-7-278

**Published:** 2014-06-19

**Authors:** Gustavo BS Rivas, Nataly Araujo de Souza, Alexandre A Peixoto, Rafaela V Bruno

**Affiliations:** 1 Laboratório de Biologia Molecular, Instituto Oswaldo Cruz, Fundação Oswaldo Cruz (FIOCRUZ), Rio de Janeiro, Brazil; 2 Laboratório de Transmissores de Leishmanioses, Instituto Oswaldo Cruz, Fundação Oswaldo Cruz (FIOCRUZ), Rio de Janeiro, Brazil; 3 Instituto Nacional de Ciência e Tecnologia em Entomologia Molecular (INCT-EM)/CNPq, Rio de Janeiro, Brazil

**Keywords:** Locomotor activity, *Lutzomyia longipalpis*, Temperature, Day length, Circadian clock

## Abstract

**Background:**

Insect vectors have been established as models in Chronobiology for many decades, and recent studies have demonstrated a close relationship between the circadian clock machinery, daily rhythms of activity and vectorial capacity. *Lutzomyia longipalpis*, the primary vector of *Leishmania (Leishmania) infantum* in the New World, is reported to have crepuscular/nocturnal activity in the wild. However, most of these studies applied hourly CDC trap captures, which is a good indicative of *L. longipalpis* behaviour, but has limited accuracy due to the inability to record the daily activity of a single insect during consecutive days. In addition, very little is known about the activity pattern of *L. longipalpis* under seasonal variations of average temperature and day length in controlled laboratory conditions.

**Methods:**

We recorded the locomotor activity of *L. longipalpis* males under different artificial regimes of temperature and photoperiod. First, in order to test the effects of temperature on the activity, sandflies were submitted to regimes of light/dark cycles similar to the equinox photoperiod (LD 12:12) combined with different constant temperatures (20°C, 25°C and 30°C). In addition, we recorded sandfly locomotor activity under a mild constant temperature (25°C with different day length regimes: 8 hours, 12 hours and 16 hours).

**Results:**

*L. longipalpis* exhibited more activity at night, initiating dusk-related activity (onset time) at higher rather than lower temperatures. In parallel, changes of photoperiod affected anticipation as well as all the patterns of activity (onset, peak and offset time). However, under LD 16:08, sandflies presented the earliest values of maximum peak and offset times, contrary to other regimes.

**Conclusions:**

Herein, we showed that light and temperature modulate *L. longipalpis* behaviour under controlled laboratory conditions, suggesting that sandflies might use environmental information to sustain their crepuscular/nocturnal activity, as well as other important aspects as mating and host-seeking at appropriate times in different seasons. Our results depict previously unappreciated aspects of the *L. longipalpis* daily rhythms of activity that might have important epidemiological implications.

## Background

*Lutzomyia longipalpis* (Diptera, Psychodidae, Phlebotominae) is the main vector of *Leishmania (Leishmania) infantum* in Latin America, broadly distributed from Mexico to Argentina [1]. In the wild, *L. longipalpis* is principally active at dusk and during the night [2,3]. Our previous results recording the locomotor activity of *L. longipalpis* under artificial conditions not only confirmed its crepuscular/nocturnal behaviour, but also suggested that blood-feeding, circadian clock and behaviour are interlinked in this insect vector [4,5]. In Brazil *L. longipalpis* is represented by a complex of cryptic species, which differ in their male mating songs, sex pheromones and molecular markers [6,7]. Hence, in the *L. longipalpis* sympatric species from Sobral, Ceará, we observed a small temporal difference in their daily activity rhythms [8], suggesting that the well-established crepuscular/nocturnal comportment in this complex might have significant temporal differences. Though our previous results [4,8] have indicated some important characteristics in *L. longipalpis* behaviour, these studies were performed under a constant mild temperature (25°C) and a light/dark regime similar to the equinox (LD 12:12; cycles of 12 h of light and 12 h of darkness). Several studies in diverse insects have exhibited the ability of behavioural entrainment to seasonal changes of temperature and photoperiod [9,10]. It is known that *L. longipalpis* and Phlebotomines in general are active throughout the year but present higher levels of density coinciding with periods of higher temperatures, humidity and rainfall [11-15]. However, these studies are mainly based on the relation between density and seasonal variations, not taking into account daily rhythms of activity. Therefore, we decided to observe the *L. longipalpis* locomotor activity patterns under controlled laboratory conditions with different temperature and photoperiods, mimicking seasonal environmental variations. Herein, we display how light and temperature are important environmental factors modulating *L. longipalpis* behaviour.

## Methods

### Insects and locomotor activity recording

**Figure 1:**
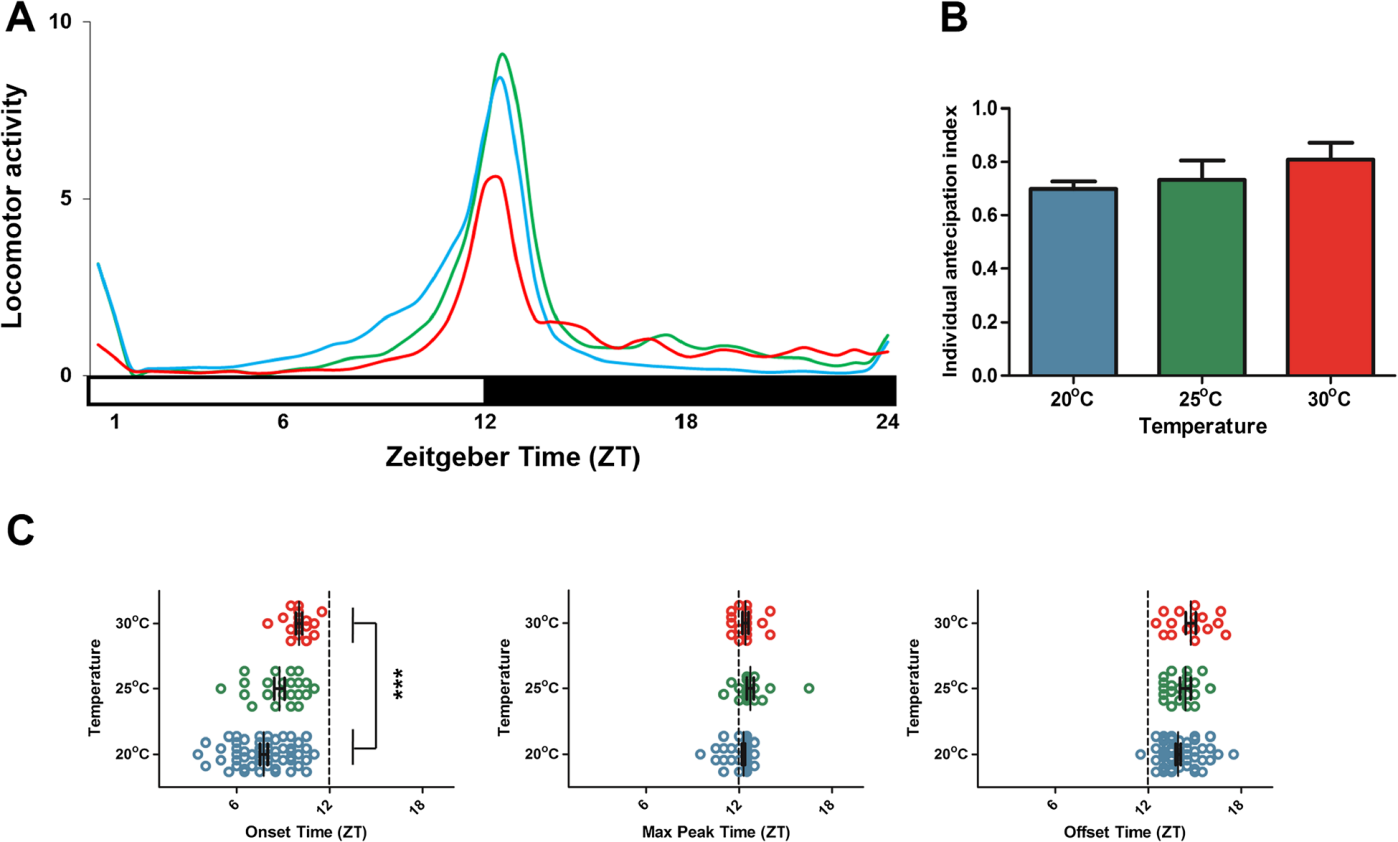
**Daily activity profile of *L. longipalpis *under LD12:12, but combined with three distinct constant temperatures. (A)** Average locomotor activity over all sandflies submitted to two days of 20°C (blue lines, *n* = 56), 25°C (green lines, *n* = 22) and 30°C (red lines, *n* = 17). The horizontal white bar indicates the light phase, while the black bar indicates the dark phase. ZT is the *Zeitgeber* time within a light/dark cycle experiment; ZT 0 corresponds to the lights-on event and ZT 12 to the lights-off event. **(B)** Mean ± SEM (vertical bars and lines, respectively) of individual anticipation index of *L. longipalpis* (see Methods) for each temperature condition. **(C)** Differences of dusk-related onset, maximum peak and offset of locomotor activity for each temperature condition. Mean ± SEM (vertical lines) and individual sandfly (circles) in *Zeitgeber* time (ZT). Lights on/off (‘dusk’) transitions are indicated by vertical dotted lines at ZT 12. ***p < 0.001; one-way ANOVA, Bonferroni’s Multiple Comparison Test (see Table 1).

**Figure 2:**
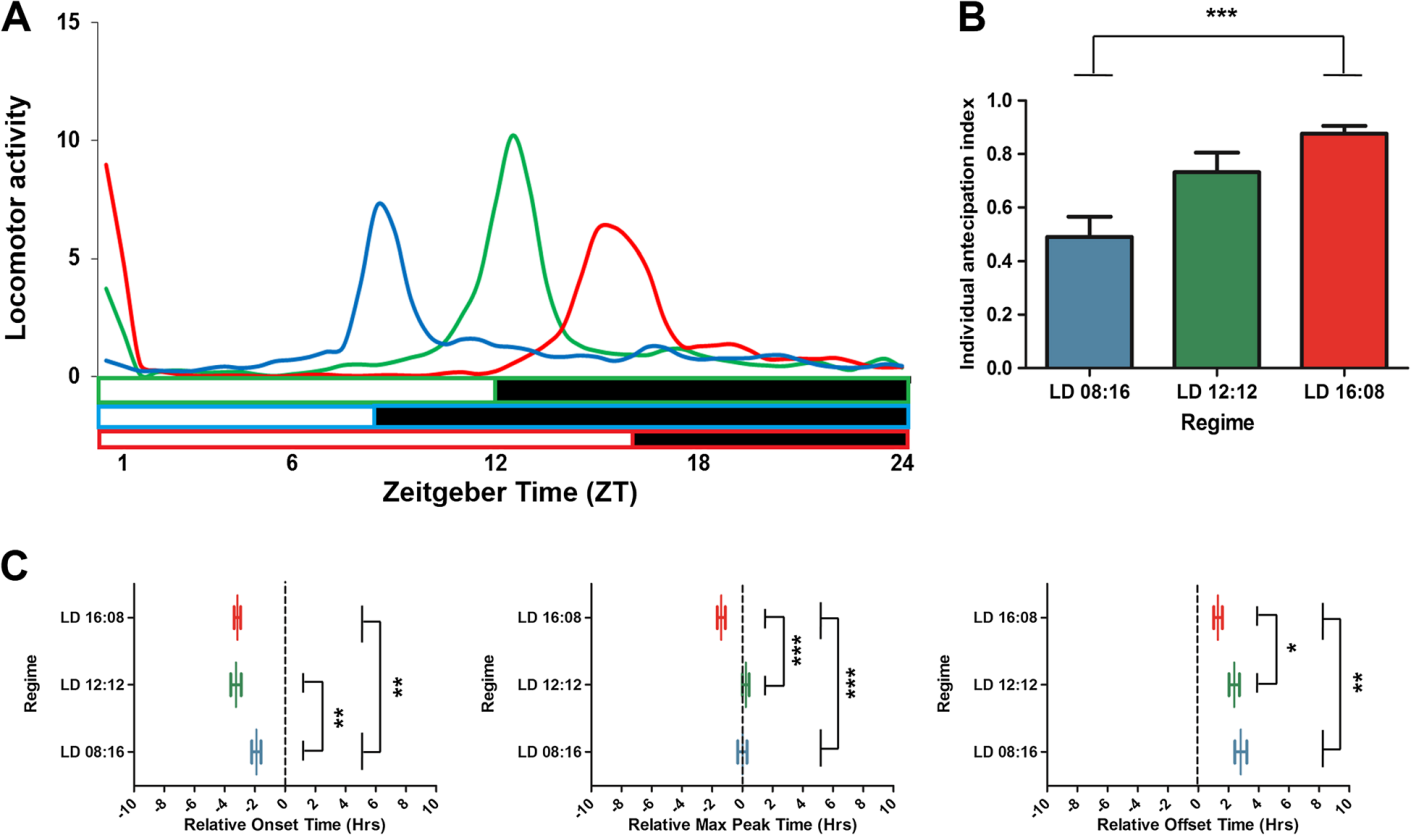
**Daily activity profile of *L. longipalpis *under 25°C, but combined with three distinct photoperiods. (A)** Average locomotor activity over all sandflies submitted to two days of LD 08:16 (blue lines, *n* = 25), LD 12:12 (green lines, *n* = 22) and LD 16:08 (red lines, *n* = 29). The horizontal white bar indicates the light phase, while the black bar indicates the dark phase. ZT is the zeitgeber time within a light/dark cycle experiment; ZT 0 corresponds to the lights-on event and ZT 8 (LD 08:16), ZT 12 (LD 12:12) and ZT 16 (LD 16:08) depict the lights-off event of each photoperiod. **(B)** Mean ± SEM (vertical bars and lines, respectively) of individual anticipation index of *L. longipalpis* (see Methods) for each photoperiod condition. **(C)** Differences of dusk-related onset, maximum peak and offset of locomotor activity for each photoperiod condition. Mean ± SEM (vertical lines) and individual sandfly (circles) in *Zeitgeber* time (ZT). Lights on/off (‘dusk’) transitions are indicated by vertical dotted lines at 0 h relative to each photoperiod (ZT 08, ZT 12 and ZT 16, see Methods for further details). *p < 0.05; **p < 0.01; ***p < 0.001; one-way ANOVA and respectively post-hoc tests (see Table 2).

*Lutzomyia longipalpis* sandflies from a Lapinha (Minas Gerais State, Brazil) laboratory colony were reared as previously described [16]. As former results from our group have demonstrated similar patterns of activity between males and females from the same colony [4], we decided to conduct our experiments with males to preserve the laboratory colony. In order to promote the appropriate entrainment for testing the effect of temperature on locomotor activity, we transferred newly emerged males to an incubator under a regime of LD 12:12, at three different constant temperatures (20°C, 25°C and 30°C) and entrained them during 2–3 days. To test the photoperiod effect on sandfly behaviour, they were entrained under a constant temperature of 25°C but combined with different photoperiods of LD 08:16 (cycles of 8 h of light and 16 h of darkness) and LD 16:08 (cycles of 16 h of light and 8 h of darkness). The choice of day length was based on several chronobiological studies in many species of insects reviewed in [9,10] allowing comparisons with previously tested photoperiodic regimes. After the entrainment, the sandflies were transferred to individual 5 mm glass tubes, which were properly sealed with plastic caps at both ends. Inside each tube we provided food ad libitum with sucrose solution soaked cotton (10%). Afterwards, we placed each tube in the Drosophila DAM5 Activity Monitoring System (Trikinetics Inc., Waltham, MA, USA) to record the locomotor activity as previously described [4,8]. When a sandfly goes back and forth within the tube, it interrupts an infrared beam that crosses the tube at its midpoint, and this interruption, detected by the onboard electronics, is added to the tube’s activity count as a measure of sandfly movement. Thus, the sandflies were monitored every 30 minutes, for five consecutive days, conserving the artificial regime previously applied in each case. However, due to the high mortality observed during the final days of the experiment, we restricted our analysis to two days valid data of the experiment. Each experiment was repeated twice and the total number of sandflies for each condition varied depending upon the mortality (Figure 1 – LD 12:12 20°C, n = 56; 25°C, n = 22; 30°C, n = 17) (Figure 2 – LD 08:16, n = 25; LD 12:12, n = 22; LD 16:08, n = 29). Average values of locomotor activity from all insects in each regime were smoothed, to reduce “noise” (abrupt variations), with a 3-point weighted moving average (weight of 2 for the central time point) and all graphs were plotted with the Excel program (Microsoft). The values depicted on graphs were also applied to estimate the absolute activity of each regime. In order to do so, we calculated the “area under the curve (AUC)” with the GraphPad Prism 5 (Prism, La Jolla, CA).

### Daily locomotor activity analysis

For our analysis, we decided to observe not only the overall locomotor activity, but also each parameter of rhythmic activity during the lights on/off transition (simulating dusk). Thus, we precisely determined the initiation of “dusk”-related activity (onset), the maximum level (peak) and the subsequent sudden drop to basal nocturnal level (offset). To do so, we determined these parameters for each insect observing the average activity of the first two experimental days with the Actogram J Program [17]. The activity onset was defined as the time of first non-zero value of activity followed by a gradual increase of it until the maximal value, which represents the peak activity. On the other hand, the offset was defined as the moment that we observed the first zero value (which depicts half an hour of inactivity) after the peak activity. In order to confirm that any advance or delay of these parameters combined would reflect on changes of anticipation, we evaluated the individual anticipation index, which is obtained by the ratio of the total amount of activity three hours before the lights off (ZT9:ZT12) over the total activity six hours before lights off (ZT6:ZT12) [18,19]. Higher values indicate high anticipation, while lower values mean little or no anticipation. In parallel, we also observed the proportion of nocturnal activity dividing the sum of activity recorded during the dark phase by the total amount of it for the whole photoperiod (24 h) [8]. We normalized data by transforming these proportional values with the arcsine square root.

### Statistical analysis

Statistical analysis and graphing were accomplished with the GraphPad Prism 5 (Prism, La Jolla, CA). We compared parameters of three groups (artificial regimes) to test the effect of temperature (20°C vs 25°C vs 30°C) and photoperiod (LD 08:16 vs LD 12:12 vs LD 16:08). One-way ANOVA depends on the assumption that the data follow a Gaussian distribution. So, we analyzed each parameter (nocturnal activity, onset time, maximum peak and offset time) with the Shapiro-Wilk normality test (p < 0.05). Only nocturnal activity and onset times passed in the normality test and were analyzed for significance with the One-Way ANOVA (Parametric test). The data of maximum peak and offset time did not pass in Shapiro-Wilk and were analyzed with the Kruskal-Wallis non-parametric test. Simultaneously, we made pairwise comparisons of regimes by two post hoc tests, the Bonferroni’s Multiple Comparison Test (parametric) and Dunn’s Multiple Comparison Test (nonparametric test).

## Results

### Daily rhythms of locomotor activity at different temperature levels

Figure 1 depicts the average activity of *L. longipalpis* at three different temperatures (20°C, 25°C and 30°C). We determined that temperature affects daily activity, considerably altering the proportion of nocturnal activity (Figure 1A and Table 1). At higher temperatures (25°C and 30°C), the sandflies are more active at night than at 20°C (Table 1, Figure 1A). Also, temperature tends to affect the startle response of maximum peak during lights on/off (1-way ANOVA; *F*_*2,94*_ = 5.847; *p* <0.01). At 30°C, the maximum peak is considerably lower compared to the other regimes (Bonferroni’s Multiple Comparison Test, 25°C vs 30°C: *t* = 3.255, *p* <0.01 and 20°C vs 30°C: *t* = 2.931, *p* <0.05).

**Table 1:** **Nocturnal activity (%), onset, maximum peak and offset time (Zeitgeber Time ± SEM) and 1-way ANOVA of the experiments depicted on Figure**1

		***Nocturnal activity***		***Onset time***		***Max peak time***		***Offset time***	
VALUES	**30°C**	62.7 ± 4.2		10 ± 0.2		12.4 ± 0.2		14.7 ± 0.3	
	**25°C**	52.3 ± 3.7		8.7 ± 0.3		12.7 ± 0.2		14.4 ± 0.3	
	**20°C**	36 ± 2.0		7.7 ± 0.2		12.3 ± 0.1		13.9 ± 0.1	
Statistical analysis	**1-way ANOVA**	*F*_2,94_ = 20.09	*P* < 0.001	*F*_2,94_ = 13.11	*P* < 0.001	*H* = 2.442^b^	*P* = 0.2949^b^	*H* = 5.636^b^	*P* = 0.0597^b^
	**25°C vs 30°C**	**NS**^**a**^		**NS**^**a**^		**NS**^**c**^		**NS**^**c**^	
	**25°C vs 20°C**	***P*** **< 0.001**^**a**^		**NS**^**a**^		**NS**^**c**^		**NS**^**c**^	
	**30°C vs 20°C**	***P*** **< 0.001**^**a**^		***P*** **< 0.001**^**a**^		**NS**^**c**^		**NS**^**c**^	

^a^Bonferroni’s Multiple Comparison Test.

^b^Kruskal-Wallis 1-way ANOVA (nonparametric test).

^c^Dunn’s Multiple Comparison Test (nonparametric test).

Although we did not find statistical differences in the anticipation index (Figure 1B), a broader peak as well as lower levels of activity during the night were evident, contrary to the pattern at 30°C (Figure 1A). Moreover, there was an earlier onset at 20°C, while these values were a bit delayed at 30°C and intermediate at 25°C (Figure 1C, Table 1). On the other hand, the peak and offset, in a first moment, seems quite similar under any temperature applied. The maximum peak was attained around half an hour after lights-off and in turn the offset time at two hours after lights-off (Figure 1C, Table 1). Nevertheless, at 20°C a number of insects peaked in their activity before lights off (Figure 1C), with a borderline statistical significance on offset time (Table 1), which suggests temperature also somewhat affects these patterns, although less than the onset time and the nocturnal activity.

### Daily rhythms of locomotor activity under different photoperiods

Next we analyzed the daily activity rhythm of *L. longipalpis* under constant temperature (25°C) and at two different photoperiodic regimes, LD 08:16 and LD 16:08, afterwards comparing them to standard conditions (LD 12:12). Evidently, *L. longipalpis* adjusts, concentrating the majority of activity during the lights on/off transition, according to the length of day (Figure 2A). Intriguingly, we observed slight reduced levels of locomotor activity under LD 08:16 (AUC_LD08:16_ = 27.06, AUC_LD12:12_ = 31.10, AUC_LD16:08_ = 30.04). Also, the sandflies exhibited considerable changes in their patterns of anticipation (Figure 2B), a result of distinct changes in their times of onset, maximum peak or offset, according to each regime (Figure 2C, Table 2). In fact, the differences of onset, peak and offset can reach as much as six hours comparing the regimes of LD 08:16 to LD 16:08 (Table 2). For a better understanding, we then plotted the difference between each value (onset, maximum peak and offset) and the light–dark transition (0 h) and noted a phase shift in all parameters that were proportional to the day length, but not exactly equal to the difference of the photoperiods (eight hours).

**Table 2:** **Onset, maximum peak and offset time (*Zeitgeber *** **Time ± SEM) and 1-way ANOVA of the experiments depicted on Figure**2

		***Onset time***		***Max peak time***		***Offset time***	
VALUES	**LD 16:08**	12.8 ± 0.2 Relative to lights off (-3.2 ± 0.2)		14.6 ± 0.2 Relative to lights off (-1.4 ± 0.2)		17.3 ± 0.3 Relative to lights off (1.3 ± 0.3)	
	**LD 12:12**	8.7 ± 0.3 Relative to lights off (-3.3 ± 0.3)		12.7 ± 0.2 Relative to lights off (0.7 ± 0.2)		14.4 ± 0.3 Relative to lights off (2.4 ± 0.3)	
	**LD 08:16**	6.1 ± 0.3 Relative to lights off (-1.9 ± 0.2)		7.9 ± 0.3 Relative to lights off (-0.1 ± 0.3)		10.8 ± 0.4 Relative to lights off (2.8 ± 0.4)	
Statistical analysis	**1-way ANOVA**	*F*_2,75_ = 6.93	*P* < 0.01	*H* = 29.43^b^	*P* < 0.001^b^	*H* = 11.22^b^	*P* < 0.01^b^
	**LD 12:12 vs LD 08:16**	***P*** **< 0.01**^**a**^		**NS**^**c**^		**NS**^**c**^	
	**LD 12:12 vs LD 16:08**	**NS**^**a**^		***P*** **< 0.001**^**c**^		***P*** **< 0.05**^**c**^	
	**LD 08:16 vs LD 16:08**	***P*** **< 0.01**^**a**^		***P*** **< 0.01**^**c**^		***P*** **< 0.01**^**c**^	

^a^Bonferroni’s Multiple Comparison Test.

^b^Kruskal-Wallis 1-way ANOVA (nonparametric test).

^c^Dunn’s Multiple Comparison Test (nonparametric test).

Curiously, there was a broader peak of locomotor activity under LD 16:08 and a narrow one under LD 08:16 (Figure 2A), suggesting different patterns of anticipation between the regimes. Even though differences of main peak and offset times compared to LD 12:12 are not apparent, under short days (LD 08:16) the sandflies displayed little anticipation (Figure 2B), initiating activity (onset time) relatively later than in other regimes (Figure 2C, Table 2). In contrast, under long days (LD 16:08) *L. longipalpis* exhibited more anticipation than in any other regime (Figure 2B). This pattern of anticipation is related to an early maximum peak, which occurred almost two hours before lights off, contrary to other regimes (Figure 2C, Table 2). Similarly, the offset time under LD 16:08 was the earliest (Figure 2C, Table 2). Thus, although *L. longipalpis* can be entrained to any photoperiod, it indicates some peculiar differences on anticipation patterns between the regimes.

## Discussion

Undoubtedly in nature, any efforts to evaluate the importance of environmental factors on insect behaviour are much more complex than under controlled laboratory conditions since these factors are multiple, oscillate simultaneously and may have distinct ranges or thresholds according to the geographical region and season. However, some important studies have been useful to better understand the influence of microclimatic influences on insect vector behavior in the field. In sandflies, both temperature and relative humidity are important influences on daily activity among different species. In Morocco, during autumn, *Phlebotomus perniciosus* has greater activity during twilight, when the humidity is low (40-50%) and the temperature high (>20°C), while *Phlebotomus ariasi* is more abundant only after midnight when humidity is high (70-80%) and temperature low (15°C) [20]. The importance of temperature and humidity for daily activity rhythms can vary depending upon the season in some sandfly species. For example, in *Lutzomyia neivai* from the province of Tucumán, Argentina, in January (summer) the nocturnal activity is mainly influenced by temperature, while in April (autumn), the humidity is the most important factor on modulation of hourly activity [21].

In the field, *L. longipalpis* has generally demonstrated a nocturnal pattern and higher levels of activity just a few hours before dusk [12,22,23], quite similar to our results presented here. Although some important studies have been establishing the effects of seasonal variations on *L. longipalpis* distribution, the consequences of seasonality on daily activity [11-15] was not explored in detail.

Our results herein, under controlled environmental conditions, showed that *L. longipalpis* tends to be active at night under higher (30°C) rather than lower temperatures (20°C), and that these patterns are mainly influenced by the time of activity increase before the lights on/off transition (onset time). However, these nocturnal activity differences are very discrete, considering only the graphs of locomotor activity, which depicts the average of several insects. Maybe the differences in the total activity of each regime and also the “smoothed” data could be decreasing the differences in the graph. Also, the time of the maximum peak of *L. longipalpis* observed here is pretty fixed to the first hour of the dark phase, regardless of the temperature applied. We speculate that abrupt changes in light on/off transitions could be fixing the maximum peak of activity proximate to ZT12 in these regimes, disguising possible temperature effects. Further experiments with gradual light on/off transitions (artificial “twilight transitions”) may permit a better visualization of the effects of temperature on maximum peak of *L. longipalpis*.

Another interesting aspect observed here was the plasticity of *L. longipalpis* to sustain their crepuscular/nocturnal activity according to the light/dark cycle duration (“long or short days”), demonstrating a seasonal adaptation of this species and a proper entrainment by light/dark cycles. A detailed analysis of these results evidenced interesting aspects of *L. longipalpis* behaviour: while in short days sandflies show little anticipation, in long days the peak and also the offset of *L. longipalpis* activity are the earliest. Interestingly in *Drosophila*, the evening peak tends to anticipate more as long as the day length increases and the possible explanation for this phenomenon rely on the circadian clock machinery [24]. It has already been well established that the molecular model of the clock has several coordinated steps over 24 hours and one of the most important is the timing of PER: TIM nuclear accumulation reviewed in [25,26]. Shafer et al. reported that the TIM nuclear accumulation is barely phase shifted by different photoperiods, while the PER nuclear accumulation phase shift depends upon the photoperiod [24]. This unusual dynamic of nuclear accumulation between PER and TIM could be affecting some particular aspects of *Drosophila* locomotor activity, such as the anticipation patterns. Despite the differences between *D. melanogaster* and *L. longipalpis* on behaviour (diurnal vs nocturnal) and feeding habits (phytophagy vs hematophagy), the circadian expression of *per* and *tim* are quite conserved between them [26]. Thus, we are tempted to speculate that the photoperiod could act similarly on clock machinery of both species and therefore, affect their behaviour. Further immunocytochemical experiments in *L. longipalpis* under different photoperiods might reveal a similar scenario on this insect vector.

Thus our results and the previous in *Drosophila* indicated that, even though both species are able to adjust to artificial photoperiodic changes, they show some deficits in the proper entrainment, which could be caused by the lack of other environmental factors, such as temperature, which fine tunes the synchronization of the circadian clock and the behaviour of these species. Indeed, when *Drosophila* is submitted to light/dark and temperature cycles, the locomotor activity and the molecular oscillation of the circadian clock both improve the entrainment [27]. Curiously, if we compare the effects of temperature with those of day-length, they are pretty complementary. *L. longipalpis* have little anticipation under LD 08:16, while at 20°C they anticipate more and have a lower proportion of nocturnal activity. On the other hand, the regime of LD 16:08 is marked by a clear anticipation pattern, while under 30°C, *L. longipalpis* has little anticipation (delayed on onset levels) and a higher proportion of nocturnal activity. Thus, considering that “winter-like” regimes are represented by short days (LD 08:16) and lower temperatures (20°C), while “summer-like” regimes by long days (LD 16:08) and higher temperatures (30°C), if we combine them according to the proper season, the overall pattern of *L. longipalpis* locomotor activity would be quite similar, which demonstrates a synergistic role of light/dark cycles and temperature to the entrainment of the circadian clock of *L. longipalpis*, as heretofore described in many insects.

Considering that *L. longipalpis* is broadly distributed from Mexico to Argentina [1] and is known as a tropical insect vector, we are led to take into account the seasonal variations of temperature as the main factor, which dictates the *L. longipalpis* daily activity rhythms in most of these regions. In fact, in the wild, there is a positive correlation between temperature and *L. longipalpis* abundance [11-15]. Herein, we also observed a positive correlation, but between temperature and nocturnal activity. According to our results, a difference of 5°C in temperature level can produce a difference of about one hour in the onset activity and a difference of 10°C can double increase the proportion of nocturnal activity. Hence, we suggest that under periods of higher temperatures, as 30°C for example, *L. longipalpis* would be not only more abundant, but also more active at night, as already reported in other sandflies during warm seasons [20,28]. However, in other regions a bit far from the equatorial zone (e.g. Yucatan Peninsula, México; N 19° or Bella Unión-Cuarein, Uruguay; S 30°), besides the differences in temperature levels, there are also differences in day length that could reach four hours from winter (10 h of daylight) to summer (14 of daylight), according to the online database of the United States Naval Observatory (USNO) Astronomy Application Department [29]. During summer (short nights) in Morocco, Guernaoui et al. described that the sandflies were active during 10 hours, whereas during autumn (longer nights), the activity extended to 14 h [20]. Our results show that a difference of four hours between different photoperiods (eg. LD 12:12 vs LD 16:08) produces a phase shift of locomotor activity patterns up to four hours of difference. Therefore, it seems plausible that, in the wild, even small variations of day length could entrain the behaviour of *L. longipalpis* and produce phase shifts in all parameters (onset, peak and offset) in the same time proportion.

Notwithstanding the well-established crepuscular/nocturnal behaviour of *L. longipalpis* in nature, previous works have reported some discrepancies in the maximum peak of activity during the night, and one of the possible reasons might be the microclimatic variations of each region [11,12,22,23]. However, it is very complex to establish the role of environmental factors on behaviour without taking into consideration many other factors such as humidity, odour, fasting, blood-intake and aging, which are interacting and contributing with this process as a whole. The fact that we evaluated daily activity of male *L. longipalpis* is interesting in some way since we could more easily avoid possible influences of insemination and blood-feeding in our results. Nevertheless, it is well known that *L. longipalpis* males are usually found in animal pens earlier than females [3,30]. Similarly, in two sympatric species from Sobral, we observed that males initiate activity somewhat earlier than females [8]. We believe that there are also sexual differences in locomotor activity of *L. longipalpis* from Lapinha [4], though it is seemingly more discrete than in sandflies from Sobral. Interestingly, although we noted small temporal differences in main activity peak among all sibling species [4,8], in each, males and females tended to conserve the time of maximum peak. Thus, our results could be a good extrapolation of what is expected for *L. longipalpis* behaviour in general, but evidently, further experiments are imperative regarding the sex and also the complex of species of this insect vector. Regardless, our results provide interesting results that reinforce some previous findings about the effects of seasonality in sandflies. Also, our individual analysis in *L. longipalpis* considering the time of onset, maximum peak and offset proved to be a reliable method for the establishment of some behavioural parameters of this insect vector. Certainly, further analyses in this sense could be helpful to better determine behavioural differences of *L. longipalpis* sex, cryptic species and vectorial capacity.

## Conclusions

In summary, *L. longipalpis* has a behavioural responsiveness to variations of temperature and photoperiod, similar to many insects, which is crucial for their physiology and ecological needs. *L. longipalpis* adjusts daily activity according to temperature, which is crucial in nature, to restricting this insect to warm temperatures, avoiding heat and desiccation. Moreover, *L. longipalpis* demonstrates a great ability of adaptation under different photoperiods sustaining the crepuscular/nocturnal activity regardless of the day length. However, under these regimes they vary in anticipation patterns, which could be due to the lack of other environmental factors, such as temperature, which acts in synergy to entrain the circadian clock and behaviour of this species. Altogether, our results can have epidemiological importance, since *L. longipalpis* is broadly distributed in Latin America, which means that they are probably exposed to different average temperature levels and somewhat different seasonal photoperiods. These differential environmental factors could be crucial to modulate not only *L. longipalpis* behaviour, but also the vectorial capacity according to the region and the season.

## Competing interest

The authors have no competing interests.

## Authors’ contribution

GBSR and AAP designed the study. GBSR performed the laboratory analyses and interpretation of data. NAS reared and provided the laboratory colony sandflies. GBSR and RVB wrote the manuscript to which all authors subsequently contributed. The final version of the manuscript was approved by all authors.
